# Reflective writing: a tool to support continuous learning and improved effectiveness in implementation facilitators

**DOI:** 10.1186/s43058-021-00203-z

**Published:** 2021-09-03

**Authors:** Tanya T. Olmos-Ochoa, Karissa M. Fenwick, David A. Ganz, Neetu Chawla, Lauren S. Penney, Jenny M. Barnard, Isomi M. Miake-Lye, Alison B. Hamilton, Erin P. Finley

**Affiliations:** 1 HSR&D Center for the Study of Healthcare Innovation, Implementation and Policy (CSHIIP), VA Greater Los Angeles Healthcare System – Sepulveda, 16111 Plummer Street (152), North Hills, CA 91343 USA; 2 David Geffen School of Medicine, University of California at Los Angeles, Los Angeles, CA USA; 3 Department of Health Policy and Management, Fielding School of Public Health, University of California at Los Angeles, Los Angeles, CA USA; 4 Elizabeth Dole Center of Excellence for Veteran and Caregiver Research, South Texas Veterans Health Care System, San Antonio, TX USA; 5 Departments of Medicine and Psychiatry, University of Texas Health Science Center at San Antonio, San Antonio, TX USA; 6 Department of Psychiatry and Biobehavioral Sciences, David Geffen School of Medicine, University of California at Los Angeles, Los Angeles, CA USA

**Keywords:** Implementation facilitation, Facilitator effectiveness, Reflective writing, Quality improvement, Qualitative methods

## Abstract

**Background:**

Implementation facilitators support the adoption of evidence-based practices and other improvement efforts in complex healthcare settings. Facilitators are trained to develop essential facilitation skills and facilitator effectiveness is typically evaluated post-implementation, but little is known about how facilitators apply and adapt didactic knowledge after training, or how learning and refining experiential knowledge occurs during the facilitation process. We propose the use of reflective writing as a tool to document and support facilitator learning and facilitator effectiveness.

**Methods:**

Using an instrumental case study of the Coordination Toolkit and Coaching (CTAC) project, we explore the use of reflective writing by facilitators to support their learning and effectiveness. Six primary care clinics participated in weekly hour-long facilitation calls over a 12-month period to implement quality improvement projects related to care coordination. Two facilitators completed templated reflections after each facilitation call for their assigned sites, totaling 269 reflections. We used the declarative-procedural-reflective model, which defines the process of skill development in clinical practice, to qualitatively analyze the reflections. Two independent coders used content analysis principles to code text that captured facilitators’ observations, evaluations, interpretations, and communication. Descriptive statistics were used to analyze reflections by facilitator and by code within and across reflections.

**Results:**

CTAC facilitators primarily used the reflections to summarize the calls (observation), assess the facilitation process and the tasks and activities they used (evaluation), document their thoughts about how to improve their own effectiveness (interpretation), and describe their communication with implementing teams. Ninety-one percent of reflections included observations, 42% interpretation, 41% evaluation, and 44% facilitator communication. In total, we coded 677 segments of text within reflections: 39% represented observation, 20% interpretation, 18% evaluation, and 23% facilitator communication.

**Conclusions:**

The process of reflective writing allowed the CTAC facilitators the time and structure to evaluate their facilitation and to think critically about how to adjust their facilitation in response to their observations and interpretations. Reflective writing is a feasible and acceptable tool to support and document facilitator learning and effectiveness.

**Trial registration:**

The project was registered with ClinicalTrials.gov (NCT03063294) on February 24, 2017.

**Supplementary Information:**

The online version contains supplementary material available at 10.1186/s43058-021-00203-z.

Contributions to the literature

- Implementation facilitators are highly skilled individuals who enable change and support improvement in complex healthcare settings. Although the skills and training required for effective facilitation have been evaluated previously, few studies have explored how to support facilitator learning and effectiveness during facilitation.
- Clinicians and other professionals use reflective writing to improve and refine their skills through observing, interpreting, and evaluating their practice. Reflective writing by facilitators may support facilitator learning, while also documenting the facilitation process in close to real time and providing additional context to evaluate facilitator effectiveness and implementation outcomes.

## Background

Implementation facilitation is an evidence-based implementation strategy used by healthcare organizations and health services researchers to support the adoption of evidence-based practices and to enable quality improvement (QI) [1, 2]. Facilitation, which often requires high-intensity interactions with healthcare staff to be successful [1], can be challenging work that entails attending to both the technical (e.g., QI methods) and relational (e.g., interpersonal dynamics) needs of the implementing staff [3, 4]. The skills needed by facilitators to effectively support implementation and QI efforts are well documented [5–8]. Less is known about whether and how the experience of facilitation impacts facilitator learning and effectiveness during the facilitation process [3, 9].

Reflection, “the process of intentionally focusing one’s attention on a particular content; observing and clarifying this focus; and using other knowledge and cognitive process to make meaningful links,” [10] has been used to enable learning within clinical and other professions [11, 12]. The declarative-procedural-reflective (DPR) model used in clinical psychology offers a comprehensive framework illustrating how reflection acts as the “engine” for learning [13], and describes the process of skill development, from didactic learning to its application and refinement in practice. Learners can engage in reflection about their interactions with clients, patients, or colleagues in the context of structured activities like supervision, consultation, and reflective writing [10, 13].

Reflective writing is defined as the practice of writing descriptively and analytically about experiences and interactions, including personal reactions and interpretations [13]. The use of reflective writing is a long-standing tradition across a variety of professions. In management, personal and unstructured reflective writing by managers can promote analysis, synthesis, and critical thinking [11]. In psychotherapy training, reflective writing can deepen skill in evidence-based practices [10]. In medical training, reflective writing through structured rubrics and creative writing exercises can improve patient care skills and provider wellbeing [14, 15].

Early evidence suggests that reflection, such as through reflective writing, may promote the development of expertise, reduce stress, prevent burnout, and increase the effectiveness in clinicians [16, 17]. Despite being distinct practices, both clinical work and implementation facilitation hinge on the application of conceptual skills and knowledge within the context of a structured interpersonal relationship. Therefore, we propose that the benefits of reflective writing seen in other fields, including skill acquisition, may also extend to implementation facilitation. Recent studies have examined how facilitators acquire and retain knowledge from trainings and how key skills are transferred from expert to novice facilitators [17, 18]. Underexplored is how facilitators adapt and refine their facilitation *during* the facilitation process and how facilitator effectiveness can be supported and sustained. Documentation of the facilitation process from the facilitator’s perspective may provide a more nuanced understanding of facilitator efforts to learn and adapt their facilitation skills and inform strategies to support and evaluate facilitator effectiveness. In this paper, we use an instrumental case study of the Coordination Toolkit and Coaching (CTAC) project in the Veterans Health Administration (VA) to describe the use of reflective writing by implementation facilitators.

## Methods

### CTAC initiative outcomes

CTAC was a QI initiative funded by the VA to improve patients’ experience of care coordination in primary care [18–20]. A cluster-randomized design was used to recruit matched pairs of VA primary care clinics assigned to either an active (distance-based facilitation plus online toolkit access) or a passive (online toolkit access only) strategy. Clinics selected locally initiated projects to address their care coordination concerns. Facilitation played a key role in helping clinic sites organize their projects to assure clinic-wide implementation, which helped improve intra-clinic communication and created hands-on experiences enabling broader QI skill development for participating staff. In contrast, clinic teams with no facilitator experienced more variable project uptake and skill development was limited to project-specific knowledge [21].

### Study design

To describe the use of reflective writing by CTAC facilitators and to better contextualize our evaluation findings, we used an instrumental case study design, which focuses more on the issue being researched (use of reflective writing) than on the case from which the issue is analyzed (CTAC) [22–25]. Data were generated by two CTAC facilitators employed to deliver the intervention; both were novice facilitators with doctoral training in health services who had reviewed a facilitation training manual developed for CTAC and shadowed a more experienced facilitator for at least 6 months prior to facilitation of CTAC sites. Each CTAC facilitator was assigned as the primary facilitator for three clinics and was responsible for hosting weekly hour-long facilitation calls with each site over a 12-month project period (*n* = 269 calls across six clinics).

**Fig. 1:**
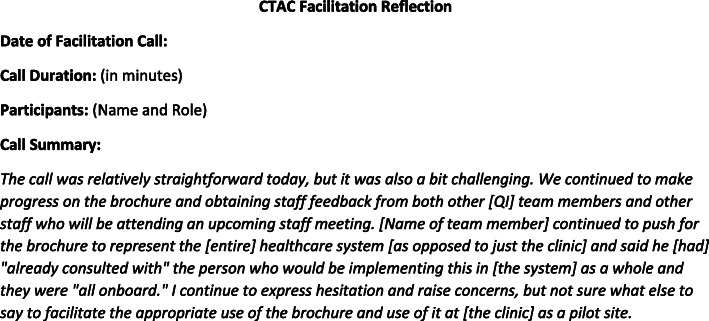
Example of CTAC facilitator reflection

At the start of facilitation, the two CTAC facilitators debriefed verbally with each other about what transpired on the initial facilitation calls; these debriefings proved helpful in thinking about the facilitation process. As a result, the two facilitators began to document and reflect on their facilitation process more consistently, with the goal of improving their facilitation over time. Facilitators logged these reflections using a simple template developed in consultation with the CTAC team, which contained prompts about the facilitation call’s date, duration, participants, an open-ended summary of what transpired on the call, and descriptions of facilitation challenges and success experienced on the call. Figure 1 provides an example of a completed facilitation reflection. Thus, in addition to hosting facilitation calls and completing site-related facilitation tasks (e.g., introducing QI methods), CTAC facilitators also completed brief (<5 min) written reflections after each facilitation call [26].

### Conceptual framework

We used the DPR model to guide our coding and analysis of facilitator reflections [10, 13]. In the DPR model, skill development and learning occur via three cognitive systems. The *declarative* system consists of conceptual, technical, and interpersonal knowledge gained from didactic training and study. In the *procedural* system, declarative knowledge is put into practice during communications with clients. Finally, the *reflective* system analyzes past, current, or future clinical experiences; compares them with stored information; identifies plans of action; and either maintains or changes stored information as a result of the analysis [13]. Thus, “information is imported into the reflective system from the declarative and procedural systems for analysis and evaluation, prior to re-export back to these systems with plans for action, change, or retention of the status quo.” [13]. Reflection is defined as “a metacognitive skill, which encompasses the *observation*, *interpretation*, and *evaluation* of one’s own thoughts, emotions and actions, and their outcomes.” [10]. Reflection through observation, interpretation, and evaluation requires focused attention on a problem, reconstruction and observation of a situation, elaboration, self-questioning, problem-solving, and imagining of alternatives [10] during and after clinical encounters. Through reflection, individuals can derive perceptual learning, or learning from a “mental representation” of events “to facilitate new understandings” that are then reinforced or debunked when applied in practice to generate new learning [13].

### Data analysis

We conducted a retrospective qualitative analysis of CTAC facilitators’ use of reflective writing during implementation. To operationalize the DPR model’s reflective system in our analysis, we generated three top-level codes representative of the reflection process: observation, evaluation, and interpretation. We defined *observation* as text in the facilitators’ reflections that was descriptive, contextual, and a neutral account of what transpired on the facilitation calls. The *evaluation* code was used to identify text that provided a general valence of the facilitation call (e.g., productive, challenging) and/or the facilitators’ self-perceived effectiveness, such as through descriptions of whether their facilitation methods were successful/unsuccessful. The *interpretation* code represented facilitators’ analyses of why events transpired as they did, along with the facilitators’ theories about how to refine their facilitation as a result of their analyses, which suggests perceptual learning or efforts to learn. Facilitators also provided examples of implementation tasks and activities that enabled them to support clinic sites (e.g., discussions related to the project timeline, providing QI methods support). We created an additional code, *facilitator communication*, to capture these tasks and activities and organize them into sub-categories in our results.

Two independent coders iteratively generated a codebook and used content analysis principles to code facilitator reflections in ATLAS.ti (version 8, GmbH, Berlin), resolving discrepancies in code application through weekly discussions to reach consensus [27, 28]. Codes were applied to complete sentences and spanned multiple sentences as needed to capture each theme occurrence. Within reflections, each code could be used more than once to capture multiple occurrences of observation, evaluation, interpretation, and facilitator communication. Following coding, the coders identified general themes and presented them to the broader CTAC team (principal investigator, project manager, project evaluator, CTAC facilitators) for discussion and further refinement [29, 30]. We used descriptive statistics to analyze reflections by facilitator and by code across sites.

## Results

CTAC facilitators’ use of reflective writing varied within and between facilitators, by length (word count), number of reflections completed per site (mean = 45), and processes logged (observation, interpretation, and evaluation, facilitator communication) (Table 1). These processes were not mutually exclusive, and reflections often contained all four.

**Table 1:** Code occurrence by reflections and coded text segments

	Observation code	Interpretation code	Evaluation code	Facilitator communication code	Total across all reflections
**Number (%) of reflections including ≥ 1 occurrence of specified code**	244 (90.7)	113 (42.0)	109 (40.5)	119 (44.2)	269 (100.0)
**Number (%) of coded text segments**	265 (39.1)	136 (20.0)	123 (18.2)	153 (22.6)	677 (100.0)

### Content of facilitator reflections

**Fig. 2:**
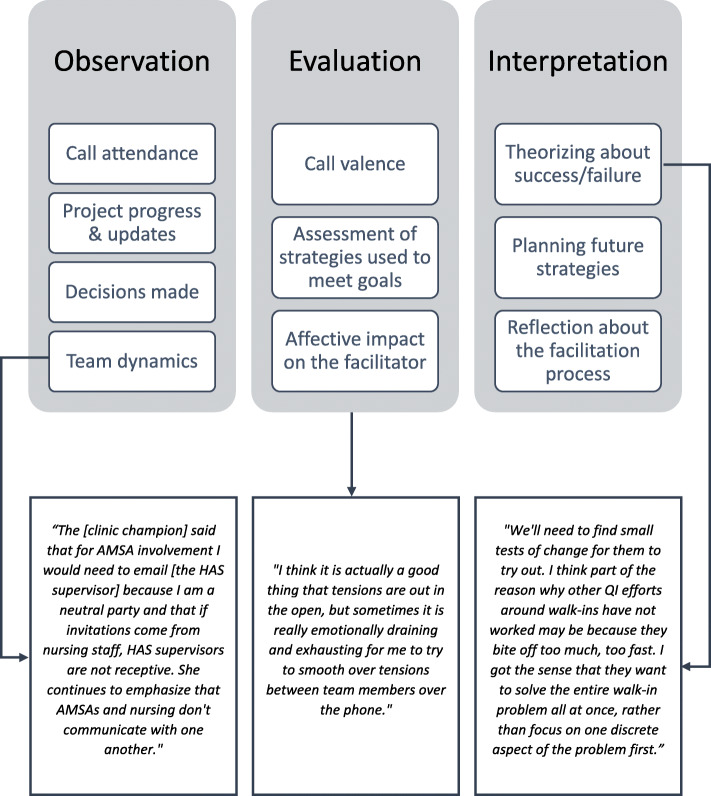
Exemplary quotes of reflective processes in facilitator reflections. Abbreviations: AMSA Advanced Medical Support Assistant, HAS Health Administration Service

The content of facilitator reflections (Fig. 2) typically started with observations that provided useful context for the facilitators’ evaluations and interpretations of the facilitation call. Observations primarily summarized the call, including descriptions of call attendance, project progress and updates, decisions made, and team dynamics. Evaluations generally focused on the perceived valence of the call (e.g., productive, challenging), facilitators’ assessment of the effectiveness of facilitation strategies used to address project goals, and the affective (e.g., mental, emotional) impact of the facilitation process on the facilitator.

Text coded as interpretation revealed the most about the facilitators’ perceptual learning and efforts to learn by documenting adaptations facilitators tested and made to their facilitation during each facilitation call. In their interpretations, facilitators reflected about their facilitation successes and challenges, including factors related to team dynamics, resistance to change, lack of participation or engagement, and project progress. Facilitators also wrote about future strategies to overcome challenges and enable success by weighing possible next steps in their facilitation. In their reflections about the facilitation process, facilitators considered the clinic environment and its impact on project progress, the implementation site’s response to QI methods and tools, and the site’s enthusiasm and engagement vis-à-vis project progress.

**Fig. 3:**
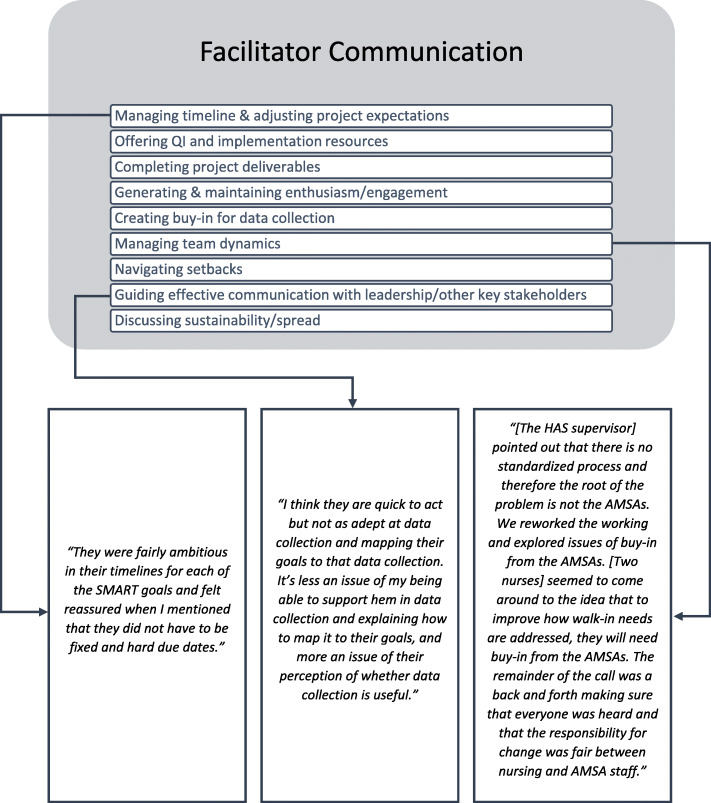
Exemplary quotes of facilitator communication in facilitator reflections. Abbreviations: AMSA Advanced Medical Support Assistant, HAS Health Administration Service, SMART Specific, Measurable, Achievable, Relevant, and Time-Bound

Descriptions of facilitators’ communication with the implementation team (Fig. 3) often occurred alongside examples of reflective interpretation and evaluation, suggesting that communication style was a frequent source of reflection, adaptation, and learning for facilitators. Facilitators communicated with sites about managing the project timeline and adjusting project expectations and suggested alternatives to elements that did not work or were outside the scope of the project. They also offered QI and implementation resources to facilitate project progress and the completion of project deliverables. Generating and maintaining enthusiasm and engagement for the project made up a significant part of the facilitators’ communication-related reflections, including maintaining momentum, encouraging attendance and verbal participation on calls, and fostering effective team communication. Facilitators also communicated with teams to create buy-in for data collection, manage team dynamics, navigate project setbacks, guide effective communication with leadership and other key stakeholders, and discuss project sustainability and spread. Additional file 1 contains exemplary quotes.

## Discussion

The process of reflection through writing allowed CTAC facilitators the time and structure to evaluate their role, to adjust their facilitation in response to their observations and interpretations, and to process the affective impact of facilitation. Reflections included observations of what transpired on the facilitation calls, evaluations of the facilitation process including facilitators’ self-perceived effectiveness, interpretations of the facilitation process including facilitators’ perceptual learning and efforts to learn, and descriptions of the facilitators’ communication.

To our knowledge, this study is the first to explore the use of reflective writing by facilitators during implementation. Existing facilitation studies report on facilitators’ characteristics and skills and on activities used by facilitators during facilitation [6–8]. However, few studies have reported on facilitators’ experiences of the facilitation process [9, 31, 32]. Reflective writing may help to capture examples of facilitator learning and self-perceived effectiveness by documenting facilitators’ application of basic didactic knowledge, perceptual learning, and the incremental development of facilitation expertise [15]. Reflective writing also enabled facilitators to continuously evaluate their facilitation process, identify areas for improvement, and support their learning and effectiveness. Reflecting via writing produced a record of facilitation activities that facilitators could later consult to recall facilitation activities and discussions. It is unclear whether reflective writing offered other unique benefits compared to alternative forms of reflection (e.g., supervision/mentoring, recordings); more work is needed to compare the potential impact of different forms of reflection on facilitator learning. Based on results, we developed and refined a sample reflective writing template with prompts designed to encourage facilitators to reflect on and document their facilitation efforts (Additional file 2).

Written reflections also provided the CTAC team and external evaluators with context to better understand the mixed-methods outcomes of the CTAC initiative [33]. For example, external evaluators reviewed the written reflections to better understand how implementing teams addressed critical junctures in the implementation process (e.g., failure/obstacles to implementation) from the facilitators’ perspectives. The reflections were helpful in providing additional context to explain trial results, assessing fidelity and adaptations to the facilitation process, documenting facilitators’ perspectives on successes and challenges to implementation, and aiding facilitator recall during weekly updates to the CTAC team. Our findings align with others suggesting that regular check-ins during implementation may improve documentation of and engagement in implementation activities [34]. Additional research is needed to assess the potential of facilitator reflections as a novel data source to evaluate facilitation and implementation outcomes.

There were several study limitations that should be considered. The CTAC reflective writing template was open-ended and relatively brief, potentially limiting the extent to which facilitators described thoughts and activities. Nonetheless, these data were rich in detail and offered insight into how facilitators reflect when given minimal prompting. CTAC facilitators were novice facilitators who chose to complete reflections; we were therefore unable to assess whether and how facilitators with different levels of training or expertise may use reflective writing differently. Furthermore, data were limited to reflections from only two facilitators, the total employed for the project. Nonetheless, the high facilitation intensity (weekly, 1-h calls over 12 months) and multiple study sites in this project resulted in a large number of written reflections that captured variations in content within and across reflections and facilitators. CTAC facilitation took place in the context of a funded QI project, and facilitators had protected time to complete their reflections (<5 min each to complete). Facilitators with higher caseloads and/or a lack of protected time may have more difficulty completing reflective writing. Finally, we did not empirically measure the relationship between reflective writing and facilitator outcomes, although the two facilitators in this study anecdotally reported that reflective writing improved their wellbeing and practice. Additional work assessing the use of templated reflections with larger facilitator samples and varying levels of facilitator expertise may address some of these limitations. Work to explore reflective writing across different facilitation settings, and in both internal and external facilitation contexts, is also needed.

## Conclusion

Two facilitators, given protected time, found reflective writing to be a feasible and acceptable tool that enabled them to document their observations, interpretations, evaluations, and communication during the facilitation process. Reflective writing provided facilitators a means by which to attend to opportunities for learning and improving their effectiveness as facilitators, while also providing an important source of real-time qualitative data on implementation progress and activities. Reflective writing by facilitators may also have potential for informing the broader study of fidelity to and outcomes in implementation facilitation.

## Supplementary Information

**Additional file 1.**

**Additional file 2.**

## Data Availability

Consents associated with primary data collection for clinician/staff participants in CTAC did not include permission to share data in publicly available repositories. De-identified administrative datasets may be eligible for future data sharing once national VA guidance on request and distribution processes are provided (in process). Final datasets will be maintained locally until enterprise-level resources become available for long-term storage and access.

## Abbreviations

CTAC: Coordination Toolkit and Coaching
QI: Quality improvement
DPR: Declarative-procedural-reflective
VA: Veterans Health Administration
AMSA: Advanced Medical Support Assistant
HAS: Health Administration Service
RN: Registered nurse
SMART: Specific, Measurable, Achievable, Relevant, and Time-Bound
