# Supplementary material for: Reflective writing: a tool to support continuous learning and improved effectiveness in implementation facilitators
Source: Implement Sci Commun. 2021 Sep 3;2:98. doi: 10.1186/s43058-021-00203-z (PMC8417958; doi:10.1186/s43058-021-00203-z)
Supplement: Supplementary file 1 — Additional file 1. [file 43058_2021_203_MOESM1_ESM.docx]

Additional file 1 Exemplary quotes of reflective processes and facilitator communication in facilitator reflections

| **Observation** | |
| --- | --- |
| **Sub-category** | **Exemplary quote** |
| *Call attendance* | Today it was just [the clinic champion] on the call, but it was nice to hear her one-on-one thoughts about the CTAC project and the work she has been doing. |
| *Project progress and updates* | On today’s call we discussed [an AMSA’s] data collection with the [other] AMSAs. He seemed to be on track with finding out how many patients they had provided the brochure to in the last week. |
| *Decisions made* | People self-assigned to two walk-in scenarios they would like to work on. |
| *Team dynamics* | One concern of mine has been that the other RNs on the call are very quiet. They don’t really speak up at all unless asked directly. When they do speak up it’s usually [RN’s Name] and the feedback is not always positive. Getting them more engaged with the project will be important. |

| **Evaluation** | |
| --- | --- |
| **Sub-category** | **Exemplary quote** |
| *Call valence* | This meeting felt like a turning point. Perhaps because they’re excited to begin a new project in their sub-committees, or because they now have one project under their belt, but the energy felt renewed. |
| *Assessing strategies used to address project goals* | We walked through the workflow…and talked about which components might make sense to adapt for their project. In the end, I'm not sure that we will revisit the workflow, but it was a good exercise nonetheless because it grounded the conversation in what their system is able to do. |
| *Affective impact of the facilitation process on the facilitator* | Today was our last call and it was definitely bittersweet. This site has been so great to coach, so dedicated to improving the quality of care at their site and such great team players. I will definitely miss coaching them. |

| **Interpretation** | |
| --- | --- |
| **Sub-category** | **Exemplary quote** |
| *Theorizing about success/failure* | I do think these two sides are hearing more of each other’s concerns now because of the coaching calls. The primary reason is that the coaching calls provide a forum for them to speak openly to a third neutral party about issues they are facing and just having the ability to hear each other’s side seems to be changing their attitudes and level of respect for one another. |
| *Planning future strategies* | We will work on a flow map next week and will have to figure out what to do to structure the project since it has the potential to be contentious between the RNs and the AMSAs. |
| *Reflection about the facilitation process* | Of my three sites, they seem to be the most efficient with the assigned time. This is probably a combination of their own team style and the fact that I have now had the benefit of coaching two sites and ironing out some of the issues with my own facilitation style. |

| **Facilitator communication** | |
| --- | --- |
| **Sub-category** | **Exemplary quote** |
| *Managing timeline and adjusting project expectations* | [Team member] wants to spread this brochure to the whole Healthcare System before we have even tested it for [this site’s] patients, and that is beyond the scope of the project at this point. Towards the end of CTAC, we can revisit the spreading of the brochure after it has been tested among [this site’s] patients. I worry a little that they could be hard to rein in, even though their enthusiasm to do this work is great. |
| *Offering QI and implementation resources* | I shared the Save-a-Trip tool from our toolkit, and everyone seemed to like it. There was additional discussion about having to update the form to reflect the needs of [the clinic’s] patients and we agreed to revisit…next week once everyone had a chance to review the form. |
| *Completing project deliverables* | We worked on the midpoint report and made good progress. |
| *Generating and maintaining enthusiasm/engagement* | I reminded him and the others that they have expressed a genuine desire to work together and improve communication between AMSA and nursing staff. |
| *Creating buy-in for data collection* | I made the point, again, that collecting the data will help them make a case for whether or not their brochure was successful. |
| *Managing team dynamics* | We are still working on the workflow implementation and smoothing over tensions between team members. We continue to talk through communication strategies like having [RN] and [HAS Supervisor] check in on a weekly basis and having nursing and MSAs jointly at meetings at least quarterly. |
| *Navigating setbacks* | Once the disruption happened, my main focus became trying to get [the Clinic Champion] to verbalize what he wanted the subcommittees to do. |
| *Guiding effective communication with leadership/other key stakeholders* | It was great to see them come together as a group and to brainstorm how best to collect this data. I thought they were very considerate of not speaking for those who were not on the call, about whether or not they would be able to complete certain tasks. |
| *Discussing sustainability/spread* | I also mentioned [our project manager] would reach out [regarding] leadership engagement which is a good conversation to have to get them thinking about sustainability and spread of their project. |

*Abbreviations*: *CTAC* Coordination Toolkit and Coaching Initiative, *AMSA* Advanced Medical Support Assistant, *HAS* Health Administration Service, *QI* quality improvement, *RN* registered nurse
