# Supplementary material for: Reflective writing: a tool to support continuous learning and improved effectiveness in implementation facilitators
Source: Implement Sci Commun. 2021 Sep 3;2:98. doi: 10.1186/s43058-021-00203-z (PMC8417958; doi:10.1186/s43058-021-00203-z)
Supplement: Supplementary file 2 — Additional file 2. [file 43058_2021_203_MOESM2_ESM.docx]

Additional file 2 Facilitator reflective writing template

Name: ____________________________

Today’s date: ______________________ Date of session: _______________

**1. Overview**

**a.** Describe the call in 2-3 words: ___________, ___________, ___________

*For example:* productive, challenging

**b.** Rate the **intensity** of the call between 1 (least intense) and 10 (most intense): _________

**c**. Why did you rate the call at this level of intensity? ________________________________

*Definition*: Intensity includes the tasks and effort involved in delivering facilitation and the mental, emotional, and physical impact on you, the facilitator, of delivering facilitation.

**d*.*** Current implementation phase: ⃝ Pre-Implementation ⃝ During ⃝ Post-Implementation

**2. Summary:** What happened during the facilitation session?

**3. Self-reflection about facilitation:** What went well? What was challenging? What are next steps?

**4. Facilitator Learning:** Which tried-and-true facilitation strategies did you use, if any? What limitations to your facilitation did you encounter? What did you do differently or adapt about your facilitation?
